# Beyond the Surface: Exploring Ancient Plant Food Processing through Confocal Microscopy and 3D Texture Analysis on Ground Stone Tools

**DOI:** 10.1007/s10816-025-09697-6

**Published:** 2025-02-08

**Authors:** Andrea Zupancich, Emanuela Cristiani, Melania Di Fazio, Laura Medeghini, Avi Gopher, Juan José Ibáñez

**Affiliations:** 1 DANTE-Diet and Ancient Technology Laboratory, Department of Oral and Maxillo Facial Sciences, Sapienza University of Rome, Rome, Italy; 2 Department of Earth Sciences, Sapienza University of Rome, Rome, Italy; 3 Sonia and Marco Nadler Institute of Archaeology, Tel Aviv University, Tel Aviv, Israel; 4 Cultural Landscapes, Milá y Fontanals Institution, Spanish National Research Council (CSIC), Barcelona, Spain

**Keywords:** GST, Confocal microscopy, 3D Surface texture analysis, Use-wear, Plants, Experimental archaeology

## Abstract

Ground stone tools are frequently found in archaeological contexts from early to late prehistoric times. These tools are key evidence for reconstructing past societies’ lifeways, technology and know-how, given their role in different tasks, including subsistence and craft activities. In recent years, the field of use-wear studies on ground stone tools showed an exponential growth in applying quantitative methods at all scales of observations, from macro to micro. These included using 3D models to measure and identify worked areas, spatial analyses for exploring trace and residue distribution, and confocal profilometers to analyse micro-polish texture. In this paper, we present results stemming from the application of confocal light microscopy and 3D surface texture analysis to identify and distinguish micro-polishes deriving from the processing of plant foods. We tested the potential of this method by analysing micro-polishes on modern limestone replicas used for grinding, pounding and dehusking wild and domestic cereal grains and legumes. Following a multi-level analysis, we demonstrate the efficacy of confocal microscopy and 3D surface texture analysis in correctly discriminating between different micro-polishes. We show how this method, integrated with the qualitative assessment of use-wear, may enhance functional interpretations of ground stone tools; we also discuss the current limits and future perspectives for their systematic application in the study of archaeological assemblages.

**Supplementary Information:**

The online version contains supplementary material available at 10.1007/s10816-025-09697-6.

## Introduction

Ground stone tools (henceforth GSTs) are one of the most ubiquitous artefacts found in archaeological contexts dated from prehistoric to historical times (de Beaune, 2004; Wright, 1992, 1994), and numerous pieces of research underlined how through their study we can enhance our understanding of various archaeological inquiries. Indeed, these tools can unveil key insights into the behavioural activities of past human groups, particularly in food preparation, due to their use in processing a wide array of organic and inorganic substances (*e.g.* plant, bone and minerals) through different tasks such as grinding, pounding, pecking, abrading and polishing (Adams, 2014; Cristiani *et al*., 2021a; de Beaune, 2004; Dietrich, 2021; Dubreuil *et al*., 2023a, 2023b; Eguíluz *et al*., 2023; Hamon, 2008; Li *et al*., 2019; Paixão *et al*., 2021; Santiago-Marrero *et al*., 2021; Zupancich *et al*., 2023; Zupancich *et al*., 2025). GSTs have provided prime evidence to explore the significance of plant foods and carbohydrate-rich diets among prehistoric communities in various world regions, including the Near East (Dietrich *et al*., 2019; Dietrich & Haibt, 2020; Dubreuil, 2004; Hayden, *et al*. 2023; Goring-Morris 2021; Santiago-Marrero *et al*., 2021), Africa (Robitaille, 2016), Western Europe (Bofill *et al*., 2013; Cristiani *et al*., 2021a; Cristiani *et al*., 2021b; Hamon, 2008; Stroulia, 2003; Stroulia *et al*., 2022; Risch, 2002, 2008; Delgado-Raack, 2013; Zimmermann, 1988; Chondrou *et al*., 2021), Asia (Li *et al*., 2019, 2020; Liu *et al*., 2010) and Australia (Fullagar *et al*., 2015; Hayes *et al*., 2022; Pardoe *et al*., 2019). Furthermore, they offer clues vis-à-vis social organisation and incipient social inequalities among prehistoric communities (Adams, 2014; Dubreuil & Plisson, 2010; Eguíluz *et al*., 2023; Stroulia *et al*., 2017; Wright, 1994).

In recent years, the field of study of functional analyses on GSTs experienced a significant growth through the development of novel methodological workflows, often focused on the application of quantitative methods (Benito-Calvo *et al*., 2018a, 2018b; Benito-Calvo *et al*., 2018a, 2018b; Caricola *et al*., 2018; Cristiani & Zupancich 2021; Delgado-Raack *et al*., 2022; Dubreuil *et al*., 2023a, 2023b; Dubreuil & Goring-Morris, 2021; Marulli *et al*., 2023; Paixão *et al*., 2021, 2022; Sorrentino *et al*., 2023; Zupancich *et al*., 2019; Zupancich & Cristiani, 2020; Chondrou* et al.*, 2021). Different approaches and equipment have been tested to effectively identify, measure and characterise use-related surface modifications at different scales. For example, close-range photogrammetry and 3D modelling in combination with spatial analyses have been successfully applied to quantify use-wear at macro- and meso-scales on experimental replicas as well as on archaeological GSTs, demonstrating their reliability in the identification of pounding, grinding and knapping gestures (Arroyo & de la Torre, 2020; Benito-Calvo *et al*., 2018a, 2018b; Caricola *et al*., 2018; Caruana *et al*., 2014). Additional studies employed confocal rugosimeter and interferometer to analyse micro-polishes and test the reliability of several surface roughness parameters in discriminating between different types of worked materials (Beyries *et al*., 1988; Bofill *et al*., 2013). For example, Bofill and colleagues (2013) showed how parameters associated with the morphology of the surface and the arithmetic mean value of the scale of the surface allow one to distinguish between tools used to process cereals and implements involved in the working of soft or greasy animal and vegetal materials (*e.g.* nuts or hide).

More recent works have used confocal microscopy to infer differences in the surface roughness of experimental GSTs used in mechanical experiments, demonstrating how several surface height and volume parameters allow the discrimination between micro-polishes associated with different organic and inorganic substances (bone, flint, nuts) (Paixão *et al*., 2021). Despite these studies, the application of confocal microscopy and surface texture quantification in GST functional studies is still in its early stages, especially when compared to knapped stone tools (*e.g.* Ibáñez *et al*., 2018; Calandra, 2022; Pedergnana *et al*., 2020; Stemp 2023, 2022, 2014; Stemp *et al*., 2013, 2015, 2009; Borel *et al*., 2021; Macdonald, 2014, 2013; Macdonald *et al*., 2020). Indeed, following the initial application of confocal microscopy and 3D surface texture analysis to distinguish between micro-polishes generated by wild and domestic cereals and reeds (Ibáñez *et al*., 2014, 2018), recent works carried out on experimental and archaeological assemblages have led to method improvements and unveiled its potentials, for example, to discriminate between different worked materials (Ibáñez *et al*., 2018; Pedergnana *et al*., 2020; Macdonald, 2013; Stemp *et al*., 2018). Further examples demonstrate how confocal microscopy and 3D surface texture analysis allow tracking changes in plant harvesting practices in SW Asia and distinguishing between cereal plant ripeness when harvested (Ibáñez-Estévez *et al*., 2021, 2025; Pichon *et al*., 2021, 2023). Also, the efficacy of confocal microscopy and 3D surface texture quantification has been tested to monitor the formation process of micro-polishes from different materials through time, providing new clues about polish formation processes and the intensity of use (Ibáñez & Mazzucco, 2021).

This paper presents the systematic application of confocal microscopy and 3D surface texture analysis to experimental GSTs used to process cereals and legumes. Our previous works (*e.g.* Cristiani & Zupancich, 2021) and experiments (see Results section), as well as recent literature (*e.g.* Adams *et al*., 2009; Dubreuil, 2001; Dubreuil *et al*., 2023a, 2023b; Hamon & Plisson, 2009; Santiago-Marrero *et al*., 2021) highlight the limits of optical microscopy to interpret micro-wear generated by cereals and legumes at a species level. Therefore, through a series of controlled experiments on modern GST limestone replicas, we explore the potential of confocal microscopy and 3D surface texture analysis to identify plant species, based on the micro-polishes generated by grinding and pounding of cereal grains and legumes.

First, we assessed the capability of this method to correctly identify used and non-used surfaces and surfaces bearing unintentional modifications (*i.e.* unintentional stone-on-stone contact) through a multi-step analysis. Subsequently, we distinguished micro-polishes associated with different species of cereal grains and legumes and activities involving their processing (*e.g.* grinding vs. dehusking/grinding vs. pounding) on a quantitative basis.

Our work demonstrates how the integration of qualitative (optical microscopy) and quantitative methods (confocal microscopy and 3D surface texture analysis) can improve the analytical resolution reaching a furtherer level of detail in our functional interpretations, representing a valuable means to understanding of GSTs use(s) in plant food processing and their significance in the lifeways of prehistoric communities.

## Materials and Methods

**Fig. 1:**
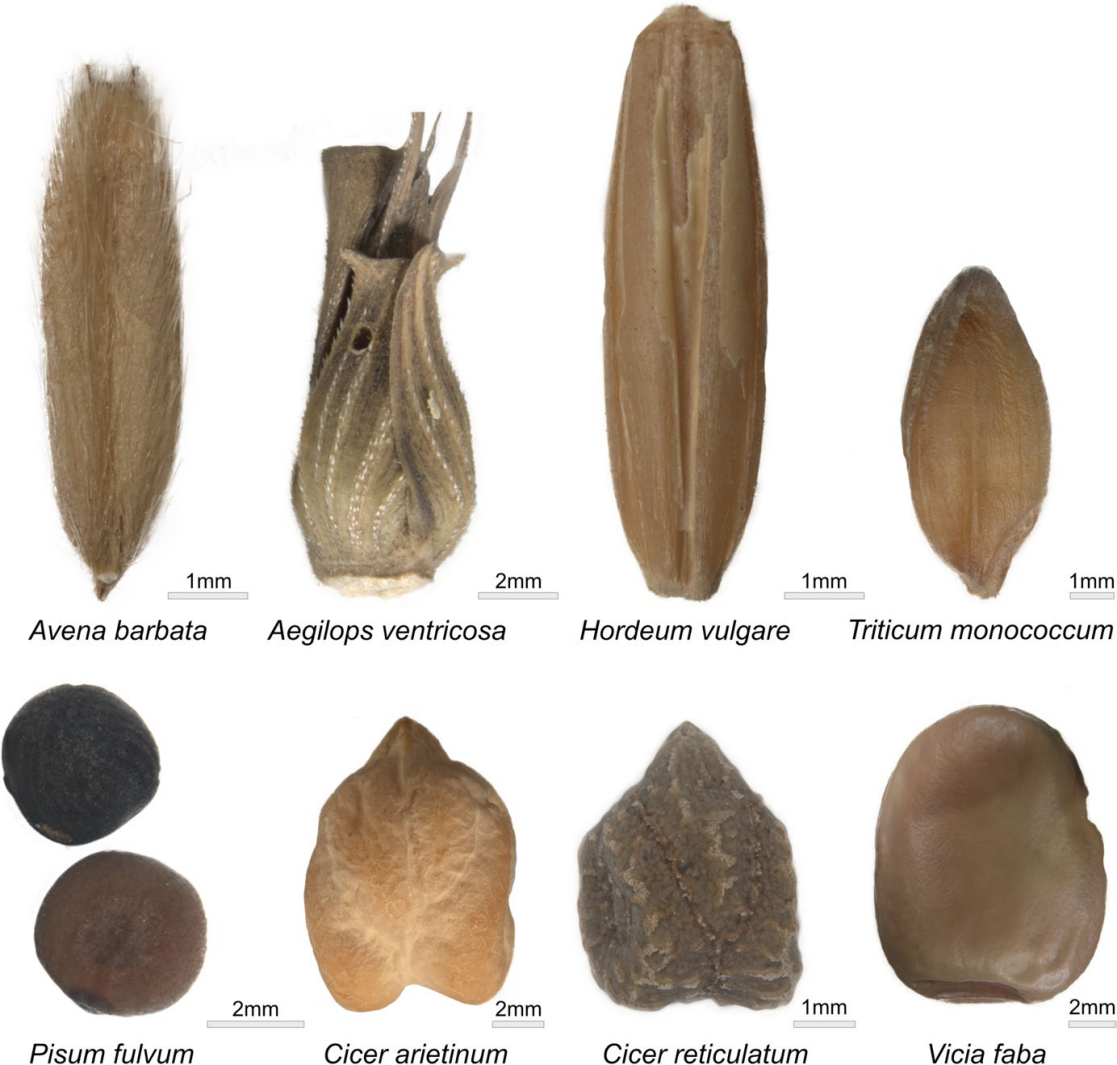
Cereal grains and legumes worked in the experimental trials

**Fig. 2:**
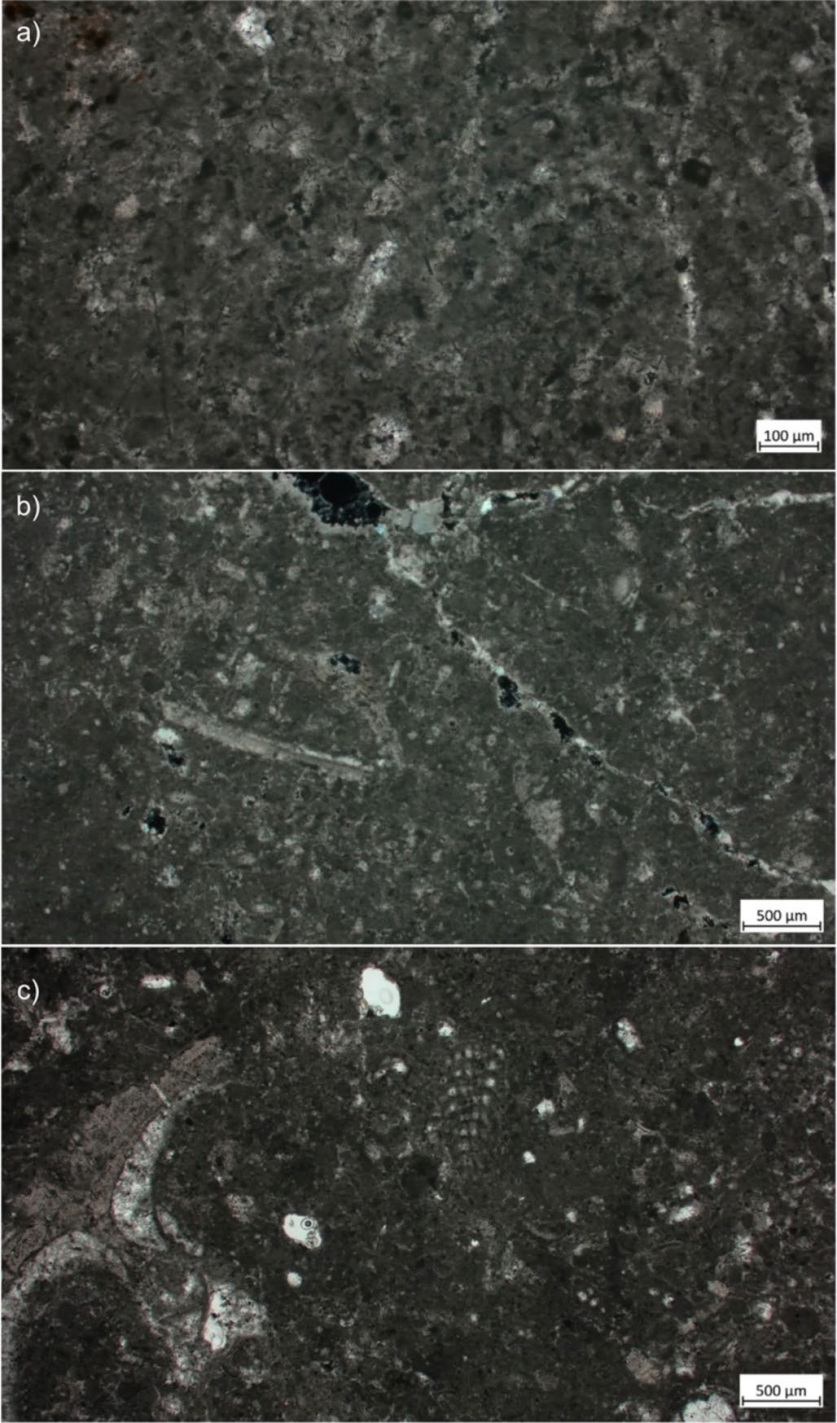
Thin section of the experimental limestone use as raw material for the GST experimental replicas. **a** Microcrystalline calcite matrix (parallel polarised light); **b** vein of calcite, inside a micritic calcite matrix (crossed polarised light); **c** microfossils of bioclasts identified inside the matrix (parallel polarised light)

Twenty modern GST replicas were used to process cereal grains and legumes, including wild and domestic species (Table 1, Fig. 1). Experimental trials (*i.e.* selection of raw material and processed plant species) were designed based on the preliminary results obtained from the use-wear and residue analysis of GSTs from three Pre-Pottery Neolithic contexts in Israel and Jordan, namely Netiv Hagdud, Nahal Yarmuth 38 and Kharaysin (Zupancich *et al*., 2023). Our results revealed that at the sites grinding slabs, mortar, pestles, and handstones were involved in the processing of a variety of plant foods. This preliminary data set the basis of our experimental framework, which comprises active and passive elements all made of limestone used to process several species of cereal grains and legumes. Limestone represents the raw material most commonly found within the investigated archaeological GST assemblages, and for this reason, it was chosen for our experimental replicas. As petrographic analyses of the archaeological limestone are currently lacking, for our experiments, we opted for a limestone of bioclastic origin, hard, compact and fine-grained collected from outcrops in central Italy, due to its similar characteristics to the archaeological one. In thin section, the limestone is matrix supported, showing an isotropic microcrystalline calcite matrix with scarce sparitic cement (Fig. 2a, b). Calcite veins are also present (Fig. 2c).

**Table 1:** Summary of the main surface modifications observed at low and high magnifications on ground stone tool replicas used to process wild and domestic cereal grains

		*Low magnifications*				*High magnifications*		
*Worked plant*	*Activity*	*Surface levelling*	*Crystal grain modification*	*Linear features*	*Surface pitting*	*Micro-polish morphology*	*Micro-polish distribution*	*Micro-striations*
Einkorn wheat (*T. monococcum*)	Grinding	Y	ND	N	N	Smooth, flat, and striated	Spot like over higher micro-topographies	Y
Oat (*A. barbata*)	Grinding	Y	Y	N	N	Smooth, pitted, and flat	Localised over the higher micro-topographies	N
Wild grass grains (*A. ventricosa*)	Grinding	Y	Y	N	N	Smooth domed	Diffused over the low and high micro-topographies	N
Wild grass grains (*A. ventricosa*)	Dehusking	N	ND	N	Y	Smooth, domed, and flat	Diffused over the low and high micro-topographies	Y
Barley (*H. vulgare)*	Grinding	Y	ND	N	N	Smooth domed	Diffused over the low and high micro-topographies	N

Y= yes; N = no; ND = indeterminable

The occurrence of allochems is evident, in particular microfossils of foraminifera and rare mollusca, from $\sim$500 μm to $\sim$1 mm (Fig. 2). The amount of allochems is $\sim$10%. Porosity is poor, with pores of micrometric size (from > 5 to $\sim$500 μm) and distributed sparsely. The pore size was estimated digitally using the interactive measurement tools available in ZEISS Zen Core (v.3.8) on the previously acquired and calibrated images. According to Folk’s classification (Folk, 1959), the rock sample can be defined as bioclastic fossiliferous micrite.

All the experimental tools are unmodified and utilised in their natural morphology, except for the two mortars, which were crafted by an expert stonecutter. In this case, modern tools were used only to shape the mortar from the limestone boulder, while its inner surface was pecked using a limestone hammer stone. For each experimental tool, before its use, a silicon cast of the non-used surface was moulded, allowing for a comparison of the non-used and used surfaces (Banks & Kay, 2003; Sorrentino *et al*., 2023). The aim of the experimental framework is twofold as it serves to (i) test the efficacy of confocal microscopy and 3D surface texture analysis in discriminating micro-polishes developed by different plant materials/activities, and (ii) build a comprehensive comparative collection of use-wear and residues associated with cereal grain and legume working using limestone ground stone tools.

To ensure comparability between the different experiments, all the activities lasted for 5h, and the processed plants were all worked in a dry state. Furthermore, at this stage of analysis, each toolset was used for a single activity and worked material. High-resolution polyvinylsiloxane (Provil Novo Light Fast Set) was used to mould casts of the used surface area, which were sampled after every hour of use (Banks & Key, 2003; Sorrentino *et al*., 2023). Before moulding, the surfaces were cleaned with hot deionised water and a 2% neutral phosphate detergent solution (Derquim) (Pedergnana and Olle, 2017).

### Optical Microscope Analysis

The used surfaces were observed at low and high magnifications following well-established methodologies (Adams *et al*., 2009; Dubreuil *et al*., 2015, 2023a, 2023b; Hamon & Plisson, 2009; Zupancich & Cristiani, 2020). A ZEISS AxioZoom V16 motorised digital stereoscope was utilised to analyse the tools at magnifications ranging from 7 × to 25 × . The extension of the used areas was assessed together with surface modification consisting of the topography of the microrelief, intergranular spaces, the morphology of the grains, macro-striation and pits (Adams *et al*., 2009; Cristiani & Zupancich 2021; Dubreuil & Savage, 2014; Hamon & Plisson, 2009). Microwear (*i.e.* micro-polish and micro-striations, abrasions, *etc*.) was observed using a ZEISS AxioScope metallographic microscope capable of magnification ranging from 100 × to 400 × . Micro-polish was identified and described according to its texture, topography, distribution and orientation, while micro-striations were described according to morphology, orientation and occurrence (Dubreuil *et al*., 2015).

### Confocal Microscope Analysis

Casts of the last stage of use (5h) of the tool were scanned using a Sensofar PluNeox white light confocal microscope. 3D images of the used surface were taken using an EPI 50X-N (0.9 NA) objective. 3D images were made of 451 planes with z-step of 0.2 µm and a threshold of 0.5%, resulting in a sampled area of 254.64 × 190.9 μm^2^. The 50 × objective was preferred to the 20 × one, usually employed in the scanning of micro-polish on knapped stone tools (*e.g.* sickle blades) (see Ibáñez *et al*., 2018; Ibáñez & Mazzucco, 2021; Pichon *et al*., 2021) as it best suits the distribution of micro-polish across the tool surface. Indeed, micro-polish on GSTs is generally scattered in spots varying in dimensions (Adams *et al*., 2009; Cristiani & Zupancich 2021; Dubreuil *et al*., 2015; Hamon & Plisson, 2009) and often interspaced by areas of the surface not affected by use. Using a 50 × objective enables sampling exclusively of the polished area, thus avoiding non-used portions that could lead to misleading observations. Ten (n 10) 3D images were took for each tool and imported in Mountains Map v.7, where sub-areas of 50 × 50 um were sampled. The numbers of sampled sub-areas varied from a minimum of 22 to a maximum of 76, for a total of 975 sub-areas (passive tools *n* = 519; active tools *n* = 446). Each sub-area was processed as follows: the topography layer was extracted, mirrored in its Z axis, levelled and the form was removed using a polynomial of degree 8. A Gaussian spatial filter (13 × 13) was applied to separate the polish texture from the irregularities of the limestone surface. Finally, a threshold operator (0.5%; 99.5%) was applied to trim the extreme values and eventual non-measured points were filled. Surface texture parameters following ISO 25178, ISO 12781 and EUR 15178N were then extracted together with data from Furrow Analysis, Particle Analysis, Fractal Analysis and Scale-Sensitive Fractal Analysis (see SI 1).

### Statistical Analysis

Texture data were statistically treated in R using RStudio (v. 2023.06.1 + 524). Micro-polish classification was performed by applying quadratic discriminant analysis to build predictive models for group membership based on Bayes’theorem standards (see Ibáñez *et al*., 2018; Ibáñez & Mazzucco, 2021; Pichon *et al*., 2021, 2023). The 68 extracted surface parameters were tested for correlation, excluding the ones showing multicollinearity (> 0.8/ =0.8). The parameters used to build the models were then selected based on Wilk's Lambda. Following published works (Ibáñez & Mazzucco, 2021; Ibáñez *et al.*, 2018), outliers greater than three times the interquartile range were replaced by the mean value. The accuracy of each model was then evaluated through blind tests. Blind tests were performed as follows: the model was trained using a training set consisting of half of the dataset, and then, the classification was performed on a test set composed of the remaining half of the micro-polish sample. The raw data and Rcode used for the analysis presented in this paper are available in Zenodo (https://doi.org/10.5281/zenodo.10949030).

## Experimental Framework

**Fig. 3:**
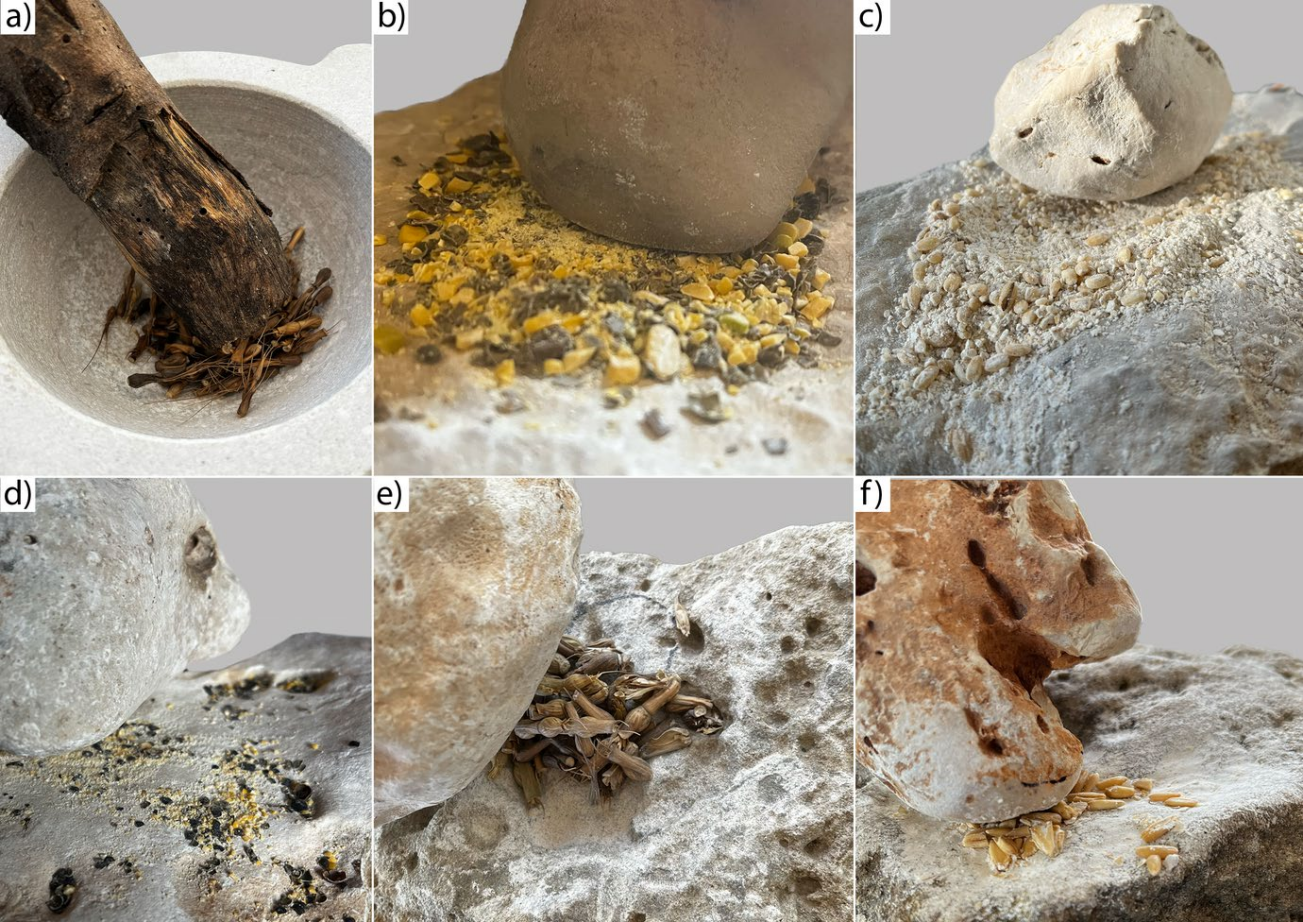
Example of some of the experimental activities performed. **a** Dehusking wild grass grains; **b** crushing and grinding wild chickpeas; **c** grinding barley; **d** crushing and grinding peas; **e** grinding wild grass grains; **f** grinding wheat

Experimental ground stone replicas were utilised to work wild and domestic species of cereal grains and legumes (Fig. 1). The experimental trials were all performed by one of the authors (AZ) and were part of a broader research project (PATH project G.A. number 101030754) focusing on the exploitation of wild and domestic plants during the Early Neolithic in the Southern Levant. The experimental activity was therefore designed based on the data available from a first analysis of the archaeological GSTs coming from the contexts studied within the PATH project. The processed plant species were selected following the archaeobotanical data available from each site and included *Triticum monococcum* (einkorn wheat), *Aegilops ventricosa* (goat grass), *Avena barbata* (oat), *Hordeum vulgare* (barley), *Cicer arietinum* (domestic chickpea), *Cicer reticulatum* (wild chickpea*)*, *Pisum fulvum* (pea), and *Vicia faba* (faba bean) (Fig. 1). The plant materials were each worked for a total of 5h in a dry state, employing motions that included back-and-forth movements (grinding), up and down motions (pounding) and mixed gestures (*i.e.* pounding and grinding) (Tables 1 and 2) (Fig. 3).

**Table 2:** Summary of the main surface modifications observed at low and high magnifications on ground stone tool replicas used to process wild and domestic legumes

		Low magnifications				High magnifications		
*Worked plant*	*Activity*	*Surface levelling*	*Crystal grain modification*	*Linear features*	*Surface pitting*	*Micro-polish morphology*	*Micro-polish distribution*	*Micro-striations*
Wild chickpea (*C. reticulatum*)	Pounding and grinding	Y	Y	N	N	Smooth, domed, and flat	Diffused over the low and high micro-topographies	N
Domestic chickpea (*C. arietinum*)	Pounding and grinding	Y	Y	N	N	Smooth, domed, and flat	Diffused over the low and high micro-topographies	N
Pea (*P. fulvum*)	Pounding and grinding	Y	Y	N	N	Smooth, domed, and flat	Diffused over the low and high micro-topographies	N
Faba bean (*V. faba*)	Pounding and grinding	Y	Y	N	Y	Smooth, domed, and flat	Diffused over the low and high micro-topographies	N
Domestic chickpea (*C. arietinum*)	Pounding	N	ND	N	Y	Smooth, domed, and flat	Diffused over the low and high micro-topographies	N

### Cereal Grains Processing

Grinding of cereal grains (*Triticum monococcum*, *Aegilops ventricosa*, *Avena barbata* and *Hordeum vulgare*) was performed through back-and-forth motions, allowing the extraction of fine flour. The cereal grains were all dehusked before being ground. One experiment was focused on dehusking wild grass grains (*Aegilops ventricosa*), which were dehusked by pounding into a limestone mortar using a pestle made of wood. The choice to use soft pestles was based on the available ethnographic and experimental literature (see Peña-Chocarro *et al*., 2009), which underlines the efficacy of wood pestles in removing the husk without damaging the grains or the mortar itself. In contrast, using a hard pestle would damage the grains, which could mix with the husk debris, thereby hindering the dehusking process (Peña-Chocarro *et al*., 2009).

### Legumes Processing

Exclusive back-and-forth movements were inefficient in obtaining flour from legumes, as the grains continuously slipped and fell from the tools’ surface. This behaviour was observed across all the processed specimens and is a result of both the shape (mostly rounded) and the hardness of the grains. For this reason, initially, the legumes were fragmented through light pounding, which facilitated their subsequent pulverisation into fine flour achieved through back-and-forth motions. Additionally, flour was obtained exclusively by pounding chickpeas using a limestone mortar and pestle. In this case, using both hard active and passive elements facilitated the fine pulverisation of the material. Of particular interest is the “flaking” observed on the inner edge of the mortar, not seen on the mortar used in dehusking, which could be considered as a further clue for identifying the use of hard pestles archaeologically.

## Results

### Optical Light Microscope (OLM)

#### Cereal Grains Processing

**Fig. 4:**
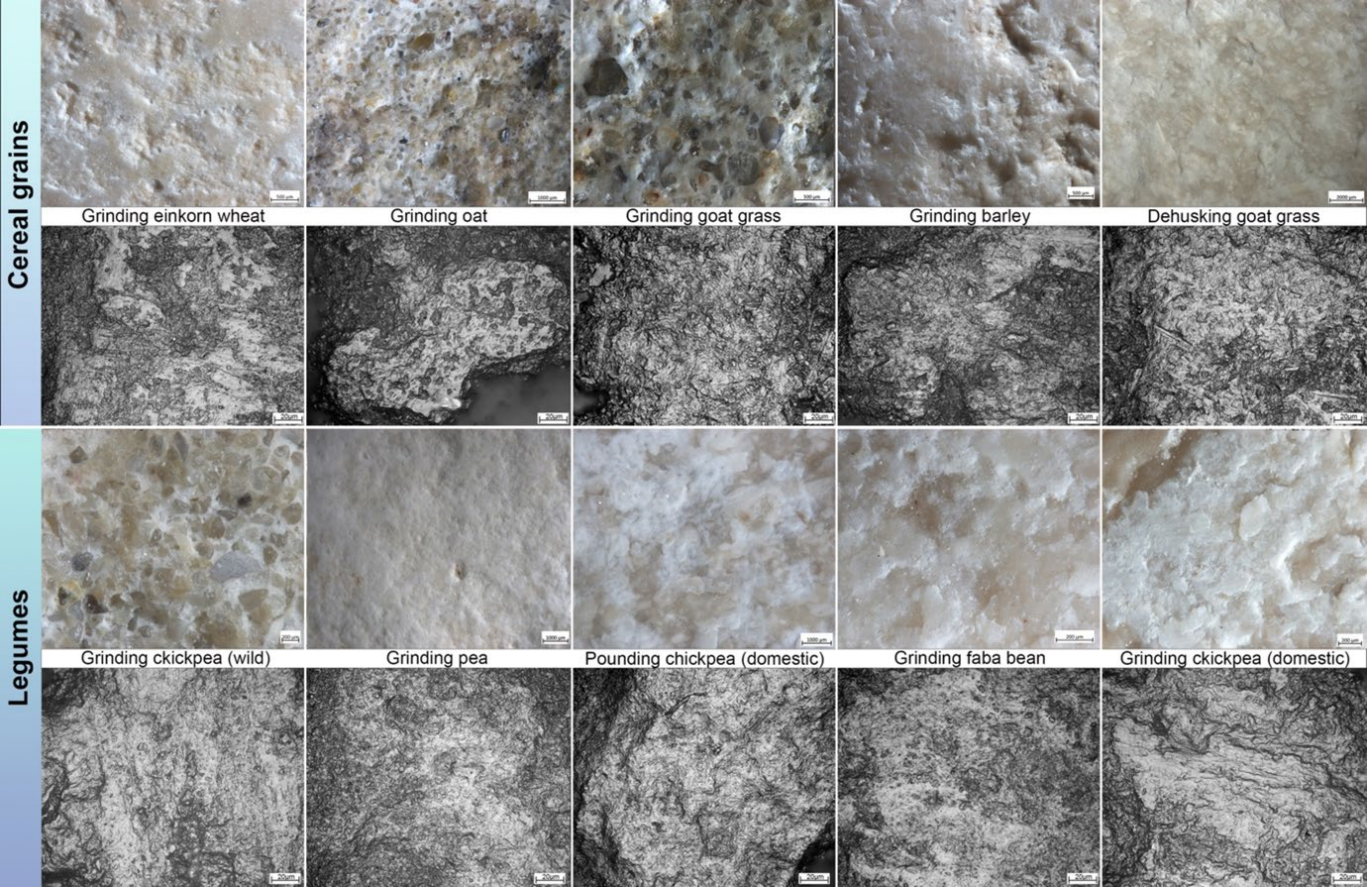
Surface modifications and micro-polishes developed on the experimental limestone ground stone tool replicas. All micro-polish pictures were taken at 400 × magnification

Grinding cereal grains (*T. monococcum*, *A. barbata*, *A. ventricosa*, *H. vulgare*) lead to the development of small to medium sized plateaus visible at low magnification across the tool’s utilised area(s). These areas exhibit a levelled and homogeneous macro topography. When present within the matrix, as in the case of the stone replicas used to grind oat and wild grass, crystal grains appear overall distinct, showing heavily rounded edges and levelled surfaces (Fig. 4). Linear features (*i.e.* macro-striae) are not visible across the surface of passive elements. At the same time, short and narrow striations are often observed across the utilised surface of active elements. At higher magnification, polished areas develop primarily over the high topographies of the micro-surface. An exception is given by the micro-polish originated by the grinding of wild grass grains and barley, which equally affects the high and low micro-topographies. In all instances, the micro-polish texture is smooth and its topography varies between the worked cereals (Fig. 4), ranging from flat and striated (einkorn wheat), reticulated (oat), domed with a greasy appearance (wild grass grains) and domed (Table 1). The distribution and orientation of polished areas vary as well. Micro-polish is spot-like and distributed across the surface of the tool used to grind einkorn wheat; it appears localised in the case of tools utilised to grind oats and diffused in the case of wild grass grains and barley. In the case of einkorn wheat, oat and barley, the micro-polish shows a clear orientation, while in the case of the tool used to grind wild grass grains, the micro-polish does not show any specific orientation.

Moreover, micro-striations are observed exclusively on the tool replica used to work einkorn wheat and appear short in length, deep and with matte bottom (Fig. 4). On the tool replica used to dehusk wild grass grains, at low magnifications, the used areas appear pitted. Pits are frequent and show an irregular morphology, while macro-striations are not visible. Microwear resulting from the dehusking of wild grass grains consists of a smooth micro-polish with a domed to flat topography. Micro-polish develops over the low and high topographies of the micro-surface, appears diffused across the used area of the tool and does not show a clear orientation. Also, wide micro-striations with a polished bottom are visible across the polished areas (Fig. 4).

### Legumes Processing

The processing of legumes (*C. arietinum*, *C. reticulatum*, *P. fulvum* and *V. faba*) through pounding and grinding gestures leads to the development of used areas characterised by small distinct plateaus and frequent grain and surface extractions visible at low magnifications. Instead, the experimental replica used to work chickpeas exclusively through pounding exhibited a used surface characterised by irregular pits and crushed areas. Crystal grains are distinct and show crushed and abraded surfaces and rounded edges (Fig. 4). Pits identified on the tool replica used to pound chickpeas are irregular and crushed surface areas are very frequent. At high magnifications, the identified micro-polish shows similar features across all the worked legumes (Table 2). Micro-polish is smooth, and its topography is domed to flat and develops over the low and high surface micro-topographies (Fig. 4). No clear difference is identified in the distribution of polished areas identified on the tool replicas, which appear highly diffused across the used surfaces. Furthermore, micro-polish exhibits a clear orientation only in the cases of wild and domestic chickpea grinding. In contrast, the micro-polish does not show a precise orientation in the other instances (*i.e.* pea and faba bean grinding and chickpea pounding).

As can be evinced from the macro and micro graphs presented in Fig. 4 as well as from the information provided in Tables 1 and 2, differences in morphology, distribution and appearance of macro- and microwear allow us to distinguish between traces generated by the processing of cereals and legumes (Adams *et al*., 2009; Dubreuil, 2001; Dubreuil *et al*., 2023a, 2023b; Hamon & Plisson, 2009; Li *et al*., 2019). However, some challenges arise when aiming to identify between different species of cereal grains and legumes. Indeed, in most cases, there are no apparent features in the distribution, invasiveness, nor the morphology of micro-polishes, which can allow for a clear and reliable inter-species identification.

### Confocal Microscopy and 3D Surface Texture Analysis

To assess the potential of confocal microscopy in enhancing the functional interpretations built through OLM, we proceeded in a “multilevel exploration” of the efficacy of 3D surface texture analysis of micro-polish: (a) discriminating between main categories of worked materials (*i.e.* plant vs. abraded stone vs. natural stone); (b) correctly identifying different subgroups within the processed plant material (*i.e.* different types of cereal grains vs legumes) and (c) discriminating within performed activities (*e.g.* grinding vs pounding vs dehusking).

### Level A: Discriminating Between Main Categories of Worked Materials

The initial level of analysis aimed to assess the efficacy of 3D surface quantification to correctly distinguish between micro-polish resulting from plant working activities, natural unused limestone surface and micro-polish produced by unintentional stone-on-stone contact. Based on a stepwise variable selection using Wilk’s lambda, 21 surface parameters were selected (Table 3).

**Table 3:** 3D surface parameters selected through Wilk’s lambda for discriminating between natural limestone surface, unintentional stone-on-stone contact, surface used to work cereals and surface used to process legumes

Name	Description	Functional group	Wilks λ	F statistics overall	F statistics diff
*Smr*	Areal material ratio	Functional	0.544363873	39.50671	39.50670921
*Str*	Texture aspect ration	Spatial	0.342412614	33.42621	27.80856033
*Svi*	Valley fluid retention index	Functional indices	0.220882153	30.87315	25.91465478
*Sdv*	Mean dale volume	Feature	0.171050556	26.21452	13.70692193
*epLsar*	Anisotropy	Direction	0.136569279	23.13402	11.86665135
*Mean density of furrows*	Mean density of furrows	Furrows	0.109328423	21.13271	11.69831349
*Sbi*	Surface bearing index	Functional indices	0.088702021	19.61845	10.90593244
*Sci*	Core fluid retention index	Functional indices	0.073684937	18.31683	9.548090092
*Sds*	Density of summits	Hybrid	0.065273202	16.86065	6.031099732
*Smr2*	Lower bearing area	Functional	0.059570987	15.53075	4.474973011
*Sal*	Fastest decay autocorrelation length	Spatial	0.055490338	14.34746	3.434225297
*Stdi*	Texture direction index	Spatial	0.05175874	13.37238	3.363277993
*Spd*	Density of peaks	Feature	0.048409381	12.54726	3.224170864
*FLTv*	Reference-to-valley flatness deviation	Flatness	0.045389204	11.83874	3.097416415
*Smr1*	Upper bearing area	Functional	0.042769506	11.21187	2.84819653
*Ssk*	Skewness	Amplitude	0.040442588	10.65592	2.672561883
*Fractal dimension*	Fractal dimension	Fractal	0.038250442	10.16922	2.659200067
*Smfc*	Scale of max complexity	Fractal	0.036108311	9.746519	2.749720281
*Smooth rough crossover*	Smooth rough crossover	Fractal	0.034487913	9.334516	2.175383973
*Sfd*	Fractal dimension of the surface	Hybrid	0.033081165	8.953622	1.966741357
*FLTp*	Peak-to-reference flatness deviation	Feature	0.031973222	8.589884	1.600931912

**Fig. 5:**
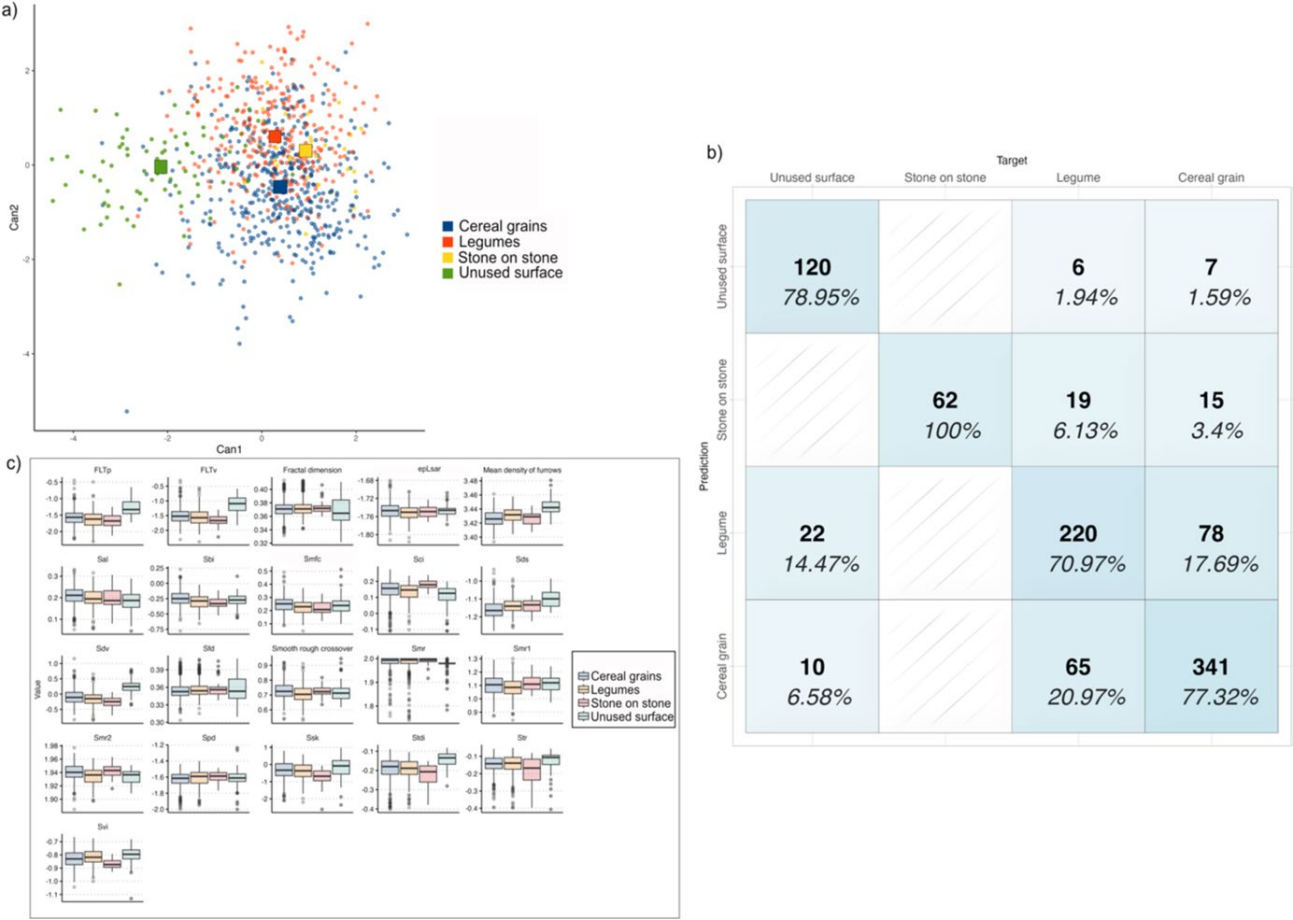
Results of the quadratic discriminant analysis to identify micro-polishes developed from the working of cereals, legumes, unintentional stone on stone contact and natural unused limestone surface. **a** Scatterplot of the first and second discriminant functions (squares indicate the group centroid); **b** confusion matrix showing the model’s accuracy in grouping each micro-polish; **c** boxplot of the 3D surface parameters used to build the model

The results of the quadratic discriminant function show a consistent classification, with 76.9% of the original groups correctly classified (Fig. 5 and Table 4).

**Table 4:** Accuracy of the model in correctly grouping micro-polish associated with cereal and legume processing, unintentional stone contact and natural limestone surface

	**Target**			
**Predicted**	Cereal grain	Legume	Unintentional stone-on-stone contact	Natural limestone
Cereal grain	**341 (77.32%)**	65 (20.97%)	0	10 (6.58%)
Legume	78 (17.69.%)	**220 (70.97%)**	0	22 (14.47%)
Unintentional stone-on-stone contact	15 (3.4%)	19 (6.13%)	**62 (100%)**	0
Natural limestone	7 (1.59%)	6 (1.94%)	0	**120 (78.95%)**
***Total analysed areas (n)***	*441*	*310*	*62*	*152*
Blind test				
Cereal grain	**156 (77.6%)**	43 (24.43%)	9 (18%)	5 (8.47%)
Legume	38 (18.9%)	**122 (69.31%)**	9 (18.32%)	4 (6.7%)
Unintentional stone-on-stone contact	0	0	**32 (64%)**	0
Natural limestone	7 (3.48%)	11 (6.25%)	0	**50 (84.74%)**
***Total analysed areas (n)***	201	176	50	59

Values in bold refer to correctly classified cases

Unused limestone surface is correctly classified in 78.95% of the cases, while each group of micro-polishes shows more than 70% of original groups correctly classified (Table 4). Micro-polish associated with cereal processing (grinding and dehusking) is successfully classified in 77.32% of the cases with a rate of misclassification lower than 20%. Specifically, 17.69% of cases were misclassified as legumes, while 1.59% of cases are incorrectly attributed to the natural limestone surface and 3.4% of specimens erroneously attributed to unintentional stone-on-stone contact. Micro-polish developed from the working of legumes (grinding and pounding) is correctly classified in 70.97% of the cases. In this case, the misclassification rate is less than 21% with 20.97% of groups misclassified as cereals, 6.13% of cases incorrectly attributed to unintentional stone-on-stone contact and 1.94% of cases misclassified as limestone unused surface. Among the analysed micro-polishes, the ones generated by unintentional contact between the two stone elements are correctly classified in all analysed cases. When a blind test is performed, the model shows an accuracy of 74.04% with all the groups correctly classified in more than 70% of the cases and an error rate of 22.53% (Table 4). The overall accuracy of the model increases when tool types are considered separately. 77.8% of the specimens are correctly grouped when only passive tools are selected, and 84.08% of successful grouping is recorded when exclusively active tools are examined.

### Level B: Discriminating Between Plant Species

#### Cereal Grains

Four different species of grains (*T. monococcum*, *H. vulgare*, *A. ventricosa* and *A. barbata*) were experimentally processed. Through the stepwise variable selection based on Wilk’s lambda, 19 surface parameters were selected (Tab 5).

**Table 5:** 3D surface parameters selected through Wilk’s lambda to distinguish between cereal grain species

Name	Description	Functional group	Wilks λ	*F* statistics overall	*F* statistics diff
*Str*	Texture-aspect ratio	Spatial	0.547268	44.67198	44.67198
*Sk*	Core roughness depth	Functional	0.305062	43.66737	42.77441
*Svi*	Valley fluid retention index	Functional indices	0.17724	42.41722	38.7636
*Sds*	Density of summits	Hybrid	0.126558	37.19462	21.47476
*Smr*	Areal material ratio	Functional	0.103329	31.9155	12.02734
*Ssk*	Skewness	Amplitude	0.085108	28.50349	11.4269
*epLsar*	Anisotropy	Direction	0.072686	25.7505	9.100672
*Smfc*	Scale of maximum complexity	Fractal	0.062437	23.71353	8.720077
*Smr1*	Upper bearing area	Functional	0.056904	21.62628	5.153974
*Sbi*	Surface bearing index	Functional indices	0.051755	20.01846	5.260286
*Sda*	Mean dale area	Feature	0.048005	18.58246	4.120781
*Sdv*	Mean dale volume	Feature	0.042626	17.71306	6.641081
*SRC threshold*	Smooth rough crossover threshold	Fractal	0.039864	16.65665	3.636573
*Smr2*	Lower bearing area	Functional	0.03731	15.75824	3.585229
*Shv*	Mean hill volume	Feature	0.035508	14.89476	2.651516
*Sha*	Mean hill area	Feature	0.032154	14.407	5.437395
*Fractal dimension*	Fractal dimension	Fractal	0.030909	13.68622	2.095186
*Svk*	Reduce valley depth	Functional	0.029682	13.05422	2.143675
*Mean density of furrows*	Mean density of furrows	Furrows	0.028618	12.47434	1.925066

**Fig. 6:**
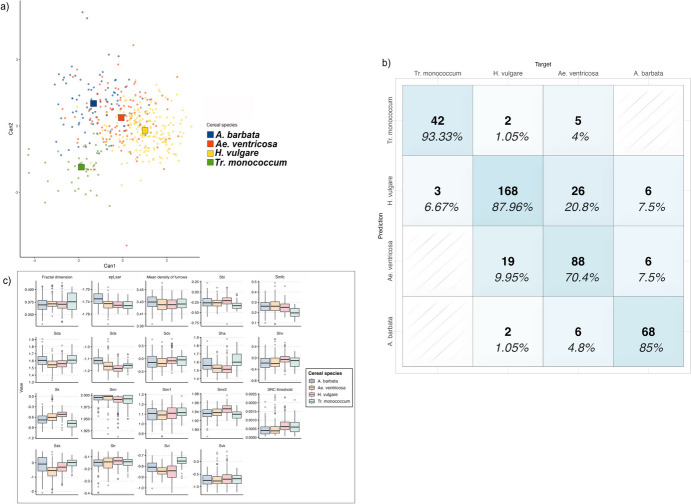
Results of the quadratic discriminant analysis to identify micro-polishes developed from the working of cereal grains. **a** Scatterplot of the first and second discriminant functions (squares indicate the group centroid); **b** confusion matrix showing the model’s accuracy in grouping each micro-polish; **c** boxplot of the 3D surface parameters used to build the model

The results of the quadratic discriminant analysis show an overall accuracy of 82.9% in correctly classifying each cereal micro-polish group (Fig. 6). All of the samples show a correct classification rate higher than 70%. Specifically, micro-polish generated by grinding einkorn wheat (93.3%) and grinding barley (87.96%) exhibits the higher rate of successful grouping, followed by micro-polishes associated with oat (85%) and wild grass grains (70.4%) (Table 6). The rate of incorrect classification is 17.23%, with the higher misclassified samples associated with micro-polishes deriving from the processing of *Aegilops ventricosa* which are incorrectly grouped as barley in 20.8% of the cases (Table 6). When blind-tested, the model returns an accuracy of 84.1% and an error rate of 15.85% (Table 6). By analysing separately active and passive tools, a significant increase in the model accuracy is recorded, with 90.3% of successful classification achieved when passive elements are considered and 97.41% of correct grouping reached by examining active tools only.

**Table 6:** Accuracy of the model in correctly identifying different cereal grains on active and passive elements. Results of the cross-validation of the model using 50% testing sample

	**Target**			
**Predicted**	*A. barbata*	*Ae. ventricosa*	*H. vulgare*	*T. monococcum*
*A. barbata*	**68 (85%)**	6 (4.8%)	2 (1.05%)	0
*A. ventricosa*	6 (7.5%)	**88 (70.4%)**	19 (9.95%)	0
*H. vulgare*	6 (7.5%)	26 (20.8%)	**168 (87.96%)**	3 (6.67%)
*T. monococcum*	0	5 (4%)	2 (1.05%)	**42 (93.3%)**
***Total analysed areas (n)***	80	123	191	45
Blind test				
*A. barbata*	**33 (86.84%)**	3 (4.34%)	5 (5.1%)	0
*A. ventricosa*	4 (10.52%)	**56 (81.15%)**	10 (10.2%)	2 (9.09%)
*H. vulgare*	1 (2.63%)	10 (14.49%)	**82 (83.6%)**	0
*T. monococcum*	0	0	1 (1.02%)	**20 (90.9%)**
***Total analysed areas (n)***	38	69	98	22

Values in bold refer to correctly classified cases

#### Legumes

Thirteen parameters were selected from the original set of 3D surface values based on Wilk’s lambda (Table 7) to discriminate between micro-polishes developed by the processing of four legume including chickpeas, peas and faba beans.

**Table 7:** 3D surface parameters selected through Wilk’s lambda to distinguish between legume species

Name	Description	Functional group	Wilks λ	*F* statistics overall	*F* statistics diff
*SRC threshold*	Smooth rough crossover threshold	Fractal	0.657593111	17.35657319	17.35657319
*Svi*	Valley fluid retention index	Functional indices	0.445499895	16.55204509	15.81640766
*epLsar*	Anisotropy	Direction	0.318281754	15.48126254	13.23460725
*Sku*	Kurtosis	Amplitude	0.257998401	13.48571038	7.710709172
*Sds*	Density of summits	Hybrid	0.214261937	12.12555088	6.713482316
*Sk*	Core roughness depth	Functional	0.191359663	10.70298834	3.922904303
*Mean density of furrows*	Mean density of furrows	Furrows	0.172534363	9.658847628	3.564274201
*Sda*	Mean dale area	Feature	0.157835694	8.815153029	3.031781484
*Smfc*	Scale of maximum complexity	Fractal	0.144813773	8.157875492	2.917464337
*Str*	Texture aspect ration	Spatial	0.132727368	7.645328957	2.944334408
*Smooth rough crossover*	Smooth, rough cross over	Fractal	0.12371825	7.164992808	2.346410528
*Sfd*	Fractal dimension of the surface	Hybrid	0.115743254	6.755885932	2.212534917
*Shv*	Mean hill volume	Feature	0.108665846	6.401086166	2.084160401

**Fig. 7:**
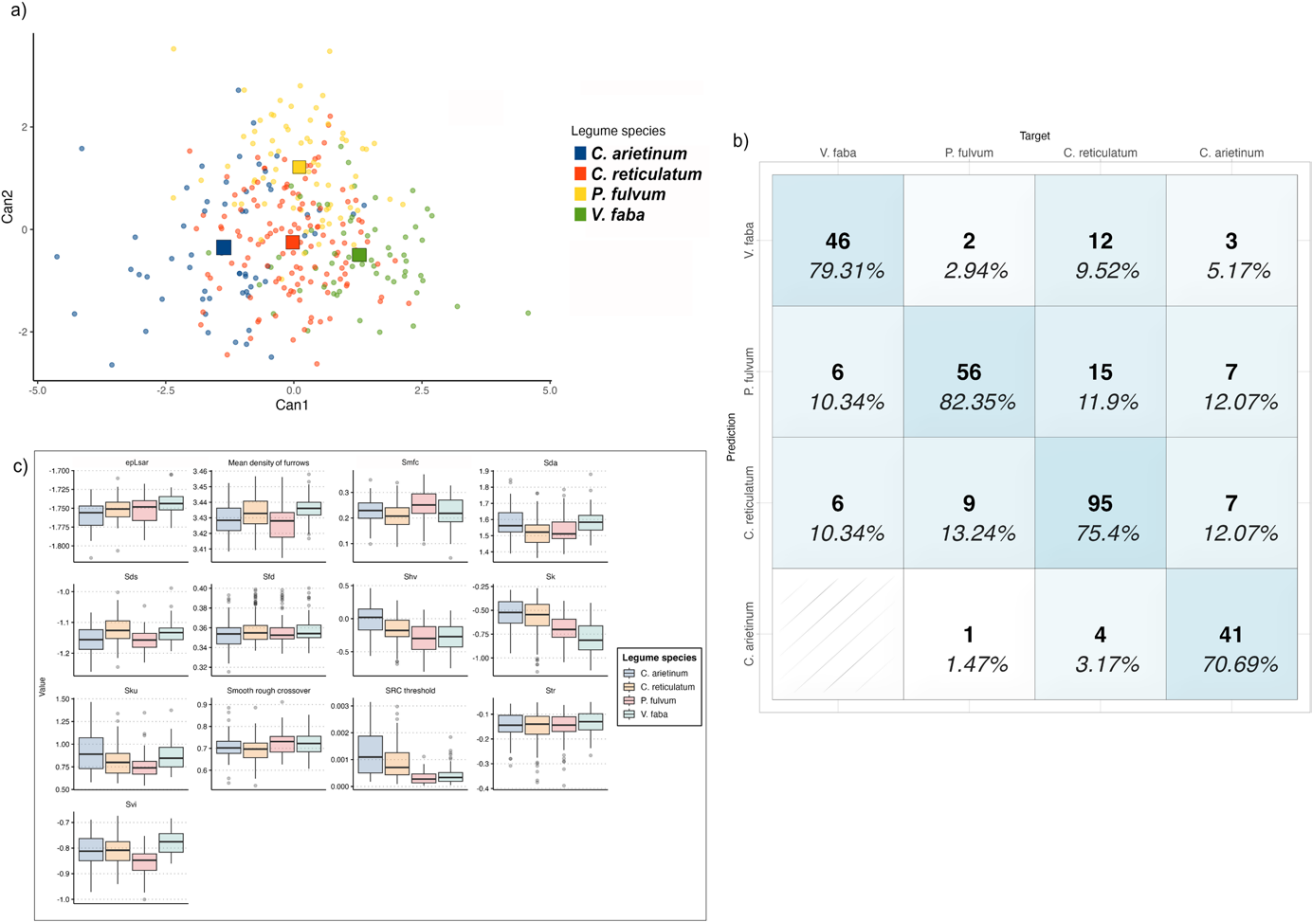
Results of the quadratic discriminant analysis to identify micro-polishes developed from the working of legumes. **a** scatterplot of the first and second discriminant functions (squares indicate the group centroid); **b** confusion matrix showing the model’s accuracy in correctly grouping each micro-polish; **c** boxplot of the 3D surface parameters used to build the model

Through quadratic discriminant analysis, 76.24% of the groups were correctly classified. Within the micro-polishes developed by the grinding of legumes, the ones associated with the processing of pea (*P. fulvum*) and faba bean (*V. faba*) exhibit the higher rate of correct classification, with 82.35% and 79.31% of successfully grouped samples (Fig. 7). Micro-polishes developed from the grinding of chickpeas show a lower degree of correct classification. Specifically, micro-polish generated by processing *C. arietinum* is correctly classified in 70.69% of the cases, with 12.07% of specimens erroneously grouped as *P. fulvum* and *C. reticulatum* respectively. Micro-polishes developed by working *C. reticulatum* are correctly grouped in 75.4% of the cases, with 11.9% of specimens incorrectly assigned as *P. fulvum* (Fig. 6 and Table 8). Blind testing the model by subsetting the sample in equal-sized train and test sets results in 75.3% of the original groups correctly classified, with an error rate of 24.6% (Table 8). As observed in the case of micro-polishes associated with the processing of cereal grains, also in this case, the accuracy of the model increases when tool types are considered independently. In particular, 85.37% of the groups are correctly classified when passive tools are examined, while 85.62% of correct grouping is achieved when analysing only active ones.

**Table 8:** Accuracy of the model in correctly identifying different cereal grains on active and passive elements. Results of the cross-validation of the model using 50% testing sample

	**Target**			
**Predicted**	*C. arietinum*	*C. reticulatum*	*P. fulvum*	*V. faba*
*C. arietinum*	**41 (70.69%)**	4 (3.17%)	1 (1.47%)	0
*C. reticulatum*	7 (12.05%)	**95 (75.4%)**	9 (13.27%)	6 (10.34%)
*P. fulvum*	7 (12.05%)	15 (11.9%)	**56 (82.35%)**	6 (10.34%)
*V. faba*	3 (5.17%)	12 (9.52%)	2 (2.94%)	**46 (79.31%)**
***Total analysed areas (n)***	58	126	68	58
Blind test				
*C. arietinum*	**23 (85.18%)**	2 (3.5%)	5 (13.15%)	1 (3.57%)
*C. reticulatum*	4 (14.81%)	**48 (84.2%)**	7 (18.42%)	7 (28%)
*P. fulvum*	0	4 (7.01%)	**26 (68.42%)**	0
*V. faba*	0	3 (5.26%)	0	**17 (68%)**
***Total analysed areas (n****)*	27	57	38	25

Values in bold refer to correctly classified cases

### Level C: Discriminating Between Activities

A final step of the analysis was testing the possibility of discriminating between micro-polishes associated with different activities carried out on the same plant. Specifically, we tested the method on micro-polish generated by wild grass grains (*A. ventricosa*) dehusking and grinding and by domestic chickpea (*C. reticulatum*) pounding and grinding. Fifteen 3D surface parameters were selected through Wilks’ lambda to build the model (Table 9).

**Table 9:** 3D surface parameters selected through Wilk’s lambda to distinguish between plant working activities

Name	Description	Functional group	Wilks λ	*F* statistics overall	*F* statistics diff
*Sdv*	Mean dale volume	Feature	0.462847609	66.15068483	66.15068483
*Mean density of furrows*	Mean density of furrows	Furrows	0.343872081	40.1141049	19.67805334
*Sci*	Core fluid retention index	Functional indices	0.284346545	29.79361131	11.88013076
*Smfc*	Scale of maximum complexity	Fractal	0.242939096	24.43869093	9.651377174
*epLsar*	Anisotropy	Direction	0.213559914	20.96392793	7.772637273
*Svi*	Valley fluid retention index	Functional indices	0.189985732	18.59527931	6.995233241
*Stdi*	Texture direction indices	Spatial	0.170216599	16.88381245	6.532933753
*Isotropy*	Isotropy	Direction	0.15529622	15.46081496	5.392315955
*Sal*	Fastest decay autocorrelation length	Spatial	0.137989905	14.60709595	7.023366008
*Smr1*	Upper bearing area	Functional	0.128972855	13.54874554	3.906462664
*Sds*	Density of summits	Hybrid	0.121738281	12.62666888	3.313070565
*Spd*	Density of peaks	Feature	0.114056678	11.9153298	3.746288243
*Fractal dimension*	Fractal dimension	Fractal	0.108399653	11.23526713	2.896363985
*Smr2*	Lower bearing area	Functional	0.102936533	10.66188335	2.938901209
*Sfd*	Fractal dimension of the surface	Hybrid	0.099650875	10.06994274	1.821686069

**Fig. 8:**
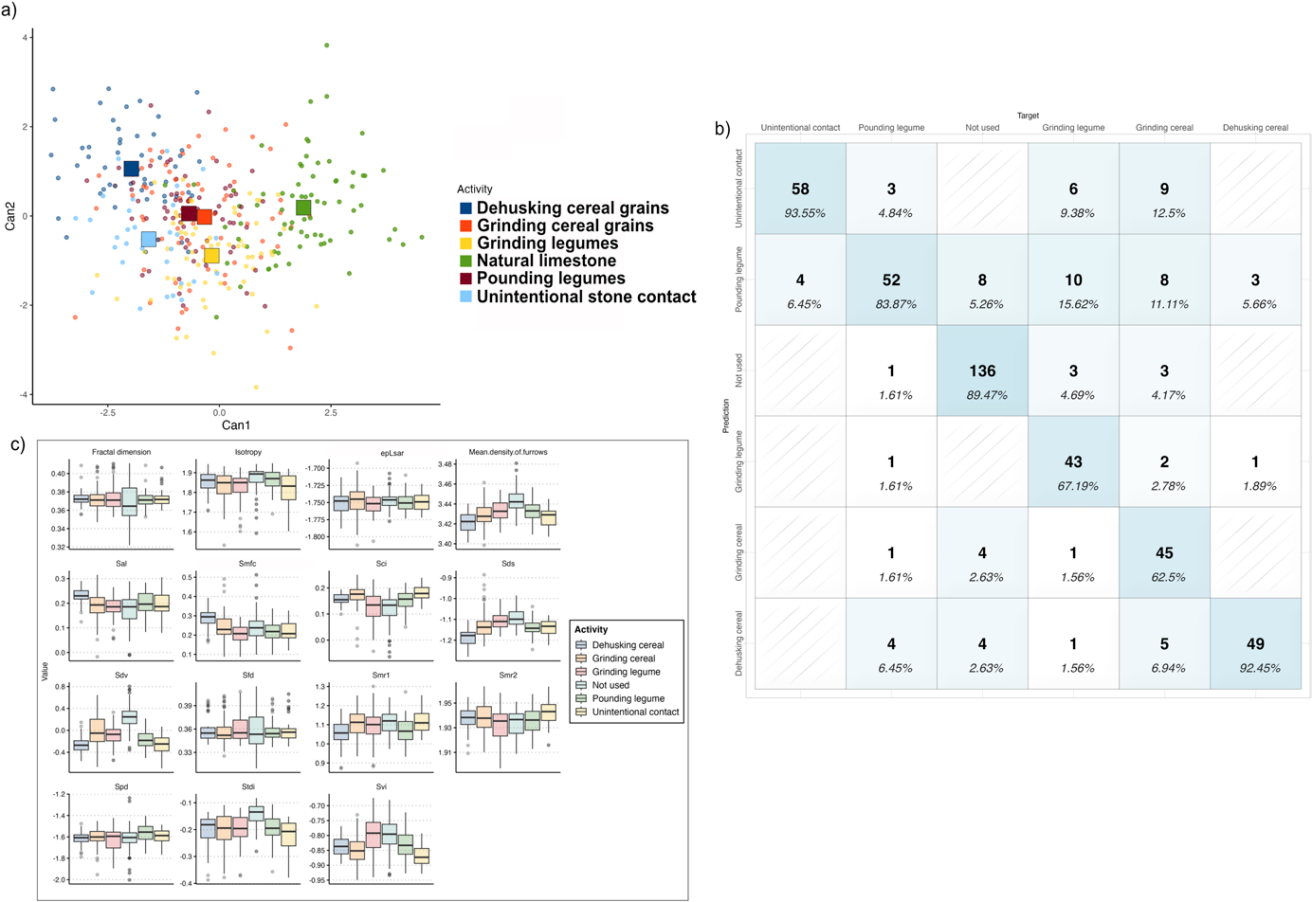
Results of the quadratic discriminant analysis to identify micro-polishes developed from grinding wild grass grains and domestic chickpeas, dehusking wild grass grains and pounding domestic chickpeas. **a** scatterplot of the first and second discriminant functions (squares indicate the group centroid); **b** confusion matrix showing the model’s accuracy in correctly grouping each micro-polish; **c** boxplot of the 3D surface parameters used to build the model

The results of quadratic discriminant analysis show an overall accuracy of 82.37% in correctly identifying the original groups. Overall, micro-polishes generated by the unintentional contact between the two stone elements are well discriminated (93.55%), as well as the non utilised limestone surfaces (89.47%). Concerning the use-related micro-polishes, the one originated by dehusking wild grass grains show the highest rate of successful classification, with 92.45% of the original groups correctly classified, followed by micro-polishes developed from the pounding of legumes among which 83.87% of the specimens are correctly classified (Fig. 8; Table 10). A lower degree of correct classification is instead noted for the micro-wear associated with the grinding of legumes and cereals, which in both cases is lower that 70% (Fig. 8; Table 10). Specifically, micro-polishes related to the grinding of chickpeas are misclassified with the micro-wear generated from the pounding of chickpeas in 15.62% of the cases, while the micro-polish generated by the grinding of wild grass grains is often misclassified as micro-wear associated with pounding legumes (11.11% of the cases) and the micro-polish developed from the unintentional stone on stone contact (12.5% of the cases). Performing a blind test, the model returns an accuracy of 81.5% and an error rate of 18.48%.

**Table 10:** Accuracy of the model in correctly identifying different activities on active and passive elements. Results of the cross-validation of the model using 50% testing sample

	**Target**					
**Predicted**	*Dehusking*	*Grinding cereal*	*Grinding legume*	*Pounding legumes*	*Natural limestone*	Unintentional stone-on-stone contact
*Dehusking*	**49 (92.45%)**	5 (6.94%)	1 (1.56%)	4 (6.45%)	4 (2.63%)	0
*Grinding cereal*	0	**45 (62.5%)**	1 (1.56%)	1 (1.61%)	4 (2.63%)	0
*Grinding legume*	1 (1.89%)	2 (4.25%)	**43 (67.18%)**	1 (1.61)	0	0
*Pounding legume*	3 (5.66%)	8 (11.1%)	10 (15.62)	**52 (83.87%)**	8 (5.26%)	4 (6.45%)
*Natural limestone*	0	3 (2.09%)	3 (4.68%)	1 (1.61%)	**136 (89.47%)**	0
Unintentional stone-on-stone contact	0	9 (12.5%)	6 (9.37%)	3 (4.83%)	0	**48 (93.54%)**
***Total analysed areas (n)***	53	72	64	62	152	62
**Blind test**						
*Dehusking*	**16 (76.19%)**	0	1 (3.57%)	3 (9.37%)	3 (4.41%)	0
*Grinding cereal*	2 (9.52%)	**23 (85.18%)**	0	1 (3.12%)	2 (2.94)	0
*Grinding legume*	0	1 (3.7%)	**16 (57.14%)**	1 (3.12%)	0	0
*Pounding legume*	1 (4.76%%)	1 (3.7%)	6 (21.4%)	**24 (75%)**	4 (5.8%)	3 (7.69%)
*Natural limestone*	2 (9.52%)	2 (7.4%)	2 (7.14%)	1 (3.12%)	**68 (88.3%)**	0
Unintentional stone-on-stone contact	0	0	3 (10.71%)	2 (6.25%)	0	**36 (92.3%)**
***Total analysed areas (n)***	21	27	28	32	77	39

Values in bold refer to correctly classified cases

However, when passive and active elements are considered separately, the model returns a higher overall accuracy. When only passive elements are considered the rate of correctly grouped micro-polishes increases to 87.83% followed by an increment in the correct classification of micro-polishes related to the grinding of legumes (84.38%) and cereals (77.14%). A similar behaviour is observed when only active elements are considered. In this case the overall accuracy of the model is 92.08%, and the correct grouping of micro-polishes generated by grinding legumes and cereals increase to 93.75% and 78.38% respectively.

## Discussion

GSTs are valuable archaeological evidence, enabling the tracing of various aspects of prehistoric communities’ lifeways. Functional analyses allow exploring past food processing strategies, dietary patterns, technological know-how and socioeconomic changes through GSTs. However, due to their typical long life cycles, characterised by multiple tasks and the processing of different kinds of organic and inorganic substances, identifying precisely the worked material presents some major challenges (Sorrentino *et al*., 2023). Consequently, in recent years, multiple workflows combining qualitative and quantitative approaches have been proposed and applied in the study of GSTs, aiming to achieve detailed, reliable and reproducible functional interpretations.

Here, we presented the systematic application of confocal microscopy and 3D surface texture quantification for analysing micro-polishes on limestone GSTs used in various plant working activities. Drawing from previous studies on knapped stone tools, we developed a workflow for acquiring 3D surface images and processing 3D texture surface parameters to explore their potential to accurately identify micro-polishes developed through processing cereal grains and legumes.

The first hypothesis tested in our multi-level study was:

- Is it possible to differentiate between used and non-used surfaces and areas affected by unintentional modifications (*i.e.* stone-on-stone contact)?

We demonstrated that quantitative measurements enable discrimination between natural limestone surfaces, micro-polishes resulting from use and those generated by accidental contact between active and passive tools. This distinction between intentional and unintentional micro-polish is crucial for analysing active elements (such as handstones used to grind cereals) and passive tools like mortars. Indeed, experimental cereal grinding revealed frequent contact between the two stones, leading to micro-polishes not directly related to the worked substance. As observed in oat grinding for example, these incidental micro-polishes may exhibit similar morphological characteristics to genuine “use-related” counterparts, posing challenges for thorough functional interpretations. Our work shows that quantitative surface measurements enable accurate assessment of a micro-polish intentional or non-intentional origin.

Once we demonstrated that microwear analysis through confocal microscopy and 3D surface texture quantification allows us to distinguish non-utilised surfaces, use and non-use-related micro-polishes, our following hypothesis was:

- Can micro-polishes developed by processing different species of cereal grains and legumes be distinguished?

Our models correctly identified most of the micro-polish samples generated by each of the worked plants, with an overall accuracy rate exceeding 75% in both cases and an inter-species correct classification rate exceeding 70%. When compared, a higher between-species accuracy (82.9% correctly grouped samples) was achieved in the analysis of micro-polishes associated with the processing of cereals compared to those related to legume processing (76.24% correctly grouped samples). These results are particularly significant because in the analysis of archaeological GSTs used in plant working activities (Cristiani *et al*., 2021a, 2021b; Hayes *et al*., 2022; Santiago-Marrero *et al*., 2021), a general interpretation of the identified microwear (*e.g.* “plant micro-polish”, “cereal micro-polish”, “legume micro-polish”) is often provided. This is due to multiple factors, including that in most cases, similar morphological features (*e.g.* texture, topography, distribution) hinder the possibility of associating a micro-polish with a given species of legume or cereal grain (see Tables 1 and 2). In this sense, integrating confocal microscopy and 3D surface texture analysis with the observation of microwear under optical light microscopes in the study of archaeological GSTs will possibly permit reaching more refined and highly detailed functional interpretations.

Finally, the last hypothesis tested through our study was:

- Do confocal microscopy and 3D surface texture analysis allow for discriminating between micro-polishes associated with different activities performed on the same worked materials?

By analysing micro-polishes developed through grinding and dehusking wild grass cereal grains and grinding and pounding legumes, we explored the possibility of discriminating between activities through 3D surface texture analysis, obtaining mixed results. While the micro-polishes related to pounding legumes and dehusking cereals are correctly identified in most instances (> 80% of the cases), the micro-polish related to grinding legumes and grinding cereals show a lower rate of correct grouping not exceeding 67.18% of cases. Concerning the grinding of legumes, we noticed a specific overlap (> 15%) with the polish generated by pounding legumes. Regarding the micro-polish generated by grinding cereals instead, we recorded an overlapping with both legume pounding (11.1%) and with micro-polish associated with the unintentional contact between the tools (12.5%) (Table 10).

Foreseeing the application of our method on GSTs from different chronologies and being aware that especially in earlier contexts these tools could have changed their role during their life cycles (*i.e.* from passive to active and *vice versa*), we performed our analyses including in the same dataset both active and passive elements. However, in all our tests, when indirectly providing the model with the information concerning the type of analysed tool, we observed a significant increase in the rate of correctly grouped micro-polishes when passive and active tools are considered separately.

Through the results presented here, we demonstrated how confocal microscopy and 3D surface texture analysis, combined with the qualitative analysis of macro and microwear, can enhance our understanding of the use of GSTs, allowing for a higher resolution in terms of micro-polish nature, worked materials and activities.

Our work contributed to the yet limited but intensively growing application of confocal microscopy and surface quantification on GSTs (Chondrou *et al*., 2021; Paixão *et al*., 2021). While this method is still in its early stages, further research is needed to comprehend its potential and limitations fully. Based on the successful application of confocal microscopy and 3D surface texture analysis on knapped stone tools, our study aimed to test if this approach would yield similar promising results on GSTs. However, the variables of our experimental trials were deliberately kept “simple” (*e.g.* one set of tools per one plant species and activity), which, while allowing for accurate testing of the approach’s potential, may not reflect the complexity observed archaeologically (Adams, 2014; Dubreuil *et al*., 2023a, 2023b).

Representing one of the first attempts to apply this method on ground stone tools, our study can be considered as a starting point which will be further refined and strengthened. Indeed, future works should test the efficacy of the methods by adding further variables, for example processing the same plant at different states (*e.g.* dry, moist) or working different substances by the same activity and gestures, which may provide answers to the question whether different materials worked in the same way would result in similar wear patterns. This will allow enhancing our proposed models, or build improved ones, expanding or reducing the number of ISO parameters, or apply new measurement approaches and further test the reliability of confocal microscopy and 3D surface texture analysis in GST studies.

Also, considering that archaeological GSTs might have been used by different members of the same community, newly designed experiments might test the possibility that the same activity performed on the same material by different individuals can result in different use-wear patterns, due to for example, the amount of pressure exerted, and how this might influence its identification through 3D surface texture analysis.

 Raw materials should also be considered in future works. GSTs are made of different lithologies, and numerous studies have highlighted how the characteristics of these materials can significantly affect the development and characteristics of micro-polish (Ibáñez & Mazzucco, 2021; Lerner *et al*., 2007; Stemp, 2014). New experimental programs are necessary to test the efficacy of this approach on other lithotypes (*e.g.* basalt and sandstone) and develop new models allowing for the application of confocal microscopy and surface texture analysis on different archaeological GST assemblages from different regions and chronologies. Finally, while our method was tested only on micro-polishes, significant functional clues can also be gathered by analysing crystal grains (Adams, 2014; Dubreuil *et al*., 2023a, 2023b, 2014; Cristiani & Zupancich 2021; Pedergnana *et al*., 2020). Therefore, future studies should focus on testing the application of confocal microscopy and surface texture analysis to effectively discriminate the range of crystal grain modification patterns associated with different materials and gestures. A further aspect to consider in the application of confocal microscopy on archaeological GSTs concerns post-depositional modifications, which at different degrees may affect the used surfaces of the tools. As demonstrated for knapped flint tools, post-depositional alteration can affect surface roughness, thus influencing the measurements of given surface parameters (Caux *et al*., 2018; Galland *et al*., 2019). Future research should address this issue for GSTs in order to explore to what extent post-depositional alterations affect 3D surface texture parameters and if they can lead to flawed functional interpretations.

The limitations mentioned above of confocal microscopy and surface texture quantification in GST functional studies prompt reflection on the application of quantitative methods in use-wear studies. In this paper, we demonstrated the potential of these methods in achieving a higher level of “functional detail”, and we believe its systematic application, along with qualitative analyses of use-wear and residues, will significantly enhance our understanding of GSTs and contribute to further insights into the field of use-wear studies. In this regard, besides serving as a powerful means for reconstructing GSTs use, our specific case study allowed for the quantitative distinction between micro-polish from different plant food species, thus enabling better reproducibility of results and data sharing—an aspect often criticised in qualitative approaches (Marreiros *et al*., 2020; Paixão *et al*., 2021). From this perspective, confocal microscopy, 3D surface texture analysis, and other quantitative approaches should not be considered a substitute for qualitative studies, as these are the foundation for thorough and reliable functional interpretations (Delgado-Raack *et al*., 2022). As emphasised in previous works, qualitative analyses are “essential in establishing the accuracy of quantitative data” (Zupancich & Cristiani, 2020 p. 10).

For example, they are crucial to identify the used area(s) on GSTs or, especially in the case of passive elements (*i.e.* grinding slabs, mortars), identify areas of the tools which can bear modification not-directly related to the processing of a given material, but rather caused by the positioning of the tool, either on the soil or on a base which can be highly informative for the reconstruction of GSTs use in the past. Also, the qualitative observations are crucial in identifying post-depositional modifications affecting the tool’s surface, which, if overlooked, can significantly compromise a functional study based solely on quantification, leading to erroneous functional interpretations. Thus, developing methodologies based on the synergetic integration of qualitative and quantitative analytical methods is essential and will pave the way for future research avenues in the field of use-wear on GSTs. This integrated approach provides opportunities to explore additional aspects of the use of these tools in the past and unveil new insights into the lifeways of past human groups.

## Conclusions

The study of ground stone tools and their use allows exploring a diverse range of aspects of the behaviour, dietary habits and lifestyle of ancient communities. These tools, ubiquitously found in archaeological contexts from different regions and of a broad chronological span starting in deep prehistory, can provide precious clues about diet, technology, know-how and social organisation of past human groups.

In recent years, the exponential growth in applying quantitative techniques in GSTs functional studies has led to the development of novel approaches involving the use of diverse methods and equipment. In this paper, we tested the application of confocal microscopy and 3D surface texture analysis to quantitatively discriminate between micro-polishes associated with plant foods processed using modern limestone GST replicas. By analysing micro-polish developed through the working of several species of cereal grains and legumes, we developed models allowing us to distinguish accurately between micro-polishes generated by actual use from those deriving from unintentional contact of passive and active tools and the natural limestone surface. We also assessed the possibility of discriminating between micro-polishes related to different species of cereal grains and legumes and between various activities. We demonstrated the high interpretative potential of confocal microscopy and 3D surface texture analysis. Still, we have underlined its limits and stressed the importance of integrating this method with qualitative use-wear analyses. Our study is one of the first attempts at applying 3D surface texture analysis on GSTs in a controlled experimental context. Future experimental works, including different raw materials and worked substances, are needed to test the potential of these methods further and foresee their systematic application in the analysis of archaeological GST assemblages.

## Supplementary Information

Below is the link to the electronic supplementary material.

Supplementary file1 (DOCX 23 KB)

## Data Availability

The dataset and R code for this paper are available in Zenodo (https://doi.org/10.5281/zenodo.10949030).
