# Supplementary material for: Beyond the Surface: Exploring Ancient Plant Food Processing through Confocal Microscopy and 3D Texture Analysis on Ground Stone Tools
Source: J Archaeol Method Theory. 2025 Feb 8;32(1):30. doi: 10.1007/s10816-025-09697-6 (PMC11807085; doi:10.1007/s10816-025-09697-6)
Supplement: Supplementary file 1 — Supplementary file1 (DOCX 23 KB) [file 10816_2025_9697_MOESM1_ESM.docx]

|  | Unit | Description of the parameter | Standard |
| --- | --- | --- | --- |
| **Amplitude Parameters** |  |  |  |
| S_q_ | *μ*m | Root mean square height | ISO 25178 |
| S_sk_ | - | Skewness | ISO 25178 |
| S_ku_ | - | Kurtosis | ISO 25178 |
| S_p_ | *μ*m | Maximum peak height | ISO 25178 |
| S_v_ | *μ*m | Maximum pit height | ISO 25178 |
| S_z_ | *μ*m | Maximum height | ISO 25178 |
| S_z_ | *μ*m | Maximum height | EUR 15178N |
| S_a_ | *μ*m | Arithmetic mean height | ISO 25178 |
| S_t_ | *μ*m | Total Height | EUR 15178N |
| **Spatial Parameters** |  |  |  |
| S_al_ | *μ*m | Fastest decay autocorrelation length | ISO 25178 |
| S_tr_ | - | Texture-aspect ratio | ISO 25178 |
| S_td_ | ° | Texture direction | ISO 25178 |
| S_td_ | ° | Texture direction | EUR 15178N |
| S_tdi_ | - | Texture direction index | EUR 16145 EN |
| **Hybrid Parameters** |  |  |  |
| S_dq_ | - | Root mean square gradient | ISO 25178 |
| S_dr_ | % | Developed interfacial area ratio | ISO 25178 |
| S_ds_ | 1/mm² | Density of summits | EUR 15178N |
| S_sc_ | 1/mm | Arithmetic mean summit curvature | EUR 15178N |
| S_fd_ | - | Fractal dimension of the surface | EUR 15178N |
| **Functional Parameters** |  |  |  |
| S_mc_ | *μ*m | Inverse areal material ratio | ISO 25178 |
| S_xp_ | *μ*m | Extreme peak height | ISO 25178 |
| S_mr_ | % | Areal material ratio | ISO 25178 |
| S_k_ | *μ*m | Core roughness depth | ISO 25178 |
| S_p_k | *μ*m | Reduced summit height | ISO 25178 |
| S_v_k | *μ*m | Reduced valley depth | ISO 25178 |
| S_mr_1 | % | Upper bearing area | ISO 25178 |
| S_mr_2 | % | Lower bearing area | ISO 25178 |
| S_pq_ | - | Plateau root mean square roughness | ISO 25178 |
| S_vq_ | - | Valley root mean square roughness | ISO 25178 |
| S_mq_ | - | Material ratio at plateau-to-valley transition | ISO 25178 |
| S_dc_ | *μ*m | Areal height difference | EUR 15178N |
| **Volume Functional Parameters** |  |  |  |
| V_m_ | mm^3^/ mm² | Material volume | ISO 25178 |
| V_v_ | mm^3^/ mm² | Void volume | ISO 25178 |
| V_mp_ | mm^3^/ mm² | Peak material volume | ISO 25178 |
| V_mc_ | mm^3^/ mm² | Core material volume | ISO 25178 |
| V_vc_ | mm^3^/ mm² | Core void volume | ISO 25178 |
| V_vv_ | mm^3^/ mm² | Pit void volume | ISO 25178 |
| **Functional Indices** |  |  |  |
| S_bi_ | - | Surface bearing index | EUR 15178N |
| S_ci_ | - | Core fluid retention index | EUR 15178N |
| S_vi_ | - | Valley fluid retention index | EUR 15178N |
| **Feature Parameters** |  |  |  |
| S_pd_ | 1/mm² | Density of peaks | ISO 25178 |
| S_pc_ | 1/mm | Arithmetic mean peak curvature | ISO 25178 |
| S_10z_ | *μ*m | Ten-point height | ISO 25178 |
| S_5p_ | *μ*m | Five-point peak height | ISO 25178 |
| S_5v_ | *μ*m | Five-point pit height | ISO 25178 |
| S_da_ | mm² | Mean dale area | ISO 25178 |
| S_ha_ |  | Mean hill area | ISO 25178 |
| S_dv_ | mm^3^ | Mean dale volume | ISO 25178 |
| S_hv_ | mm^3^ | Mean hill volume | ISO 25178 |
| **Flatness Parameters** |  |  |  |
| F_LTt_ | - | Peak-to-valley flatness deviation of the surface | ISO 12781 |
| F_LTp_ | *μ*m | Peak-to-reference flatness deviation | ISO 12781 |
| F_LTv_ | *μ*m | Reference-to-valley flatness deviation | ISO 12781 |
| F_LTq_ | *μ*m | Root mean square flatness deviation | ISO 12781 |
| **Furrows Analysis** | | | |
| Maximum depth of furrows | *μ*m | Maximum depth of furrows |  |
| Mean depth of furrows | *μ*m | Mean depth of furrows |  |
| Mean density of furrows | *mm/mm^2^* | Mean density of furrows |  |
| **Scale Sensitive Fractal Analysis (SSFA)** | | | |
| Fractal dimension | - | The range of scales in which the slope of the line is straight | ISO 25178-2 |
| Fractal dimension (Dls) | - | The range of scales in which the slope of the line is straight | ISO 25178-2 |
| Fractal complexity (Lsfc / Asfc) | *-* | How much of the surface is more complex than a Euclidean plane | ISO 25178-2 |
| Smooth-rough crossover | *-* | Scale of the smooth‐rough crossover | ISO 25178-2 |
| SRC Threshold | *-* | Threshold relative length of the smooth‐rough crossover | ISO 25178-2 |
| Scale of max complexity (Smfc) | *μ*m | Sale where highest complexity is found | ISO 25178-2 |
| Length-scale anisotropy (epLsar) | *-* | Texture anisotropy | ISO 25178-2 |
| **Texture direction** |  |  |  |
| Isotropy | *%* | Texture direction | ISO 25178-2 |
| **Other 3D Parameters** |  |  |  |
| S_mean_ | *μ*m | Mean height in absolute |  |
| S_dar_ | mm² | Developed area |  |
| S_par_ | mm² | Projected area |  |
| S_Wt_ | *μ*m | Area waviness height | ASME B46.1 |

**SI table 1.** 3D surface parameters measured using Mountains Map v.7
